# Pilose Antler Peptide-3.2KD Ameliorates Adriamycin-Induced Myocardial Injury Through TGF-β/SMAD Signaling Pathway

**DOI:** 10.3389/fcvm.2021.659643

**Published:** 2021-05-28

**Authors:** Yan Xu, Xiaobo Qu, Jia Zhou, Guangfu Lv, Dong Han, Jinlong Liu, Yuexin Liu, Ying Chen, Peng Qu, Xiaowei Huang

**Affiliations:** ^1^School of Pharmaceutical, Changchun University of Chinese Medicine, Changchun, China; ^2^Jilin Ginseng Academy, Changchun University of Chinese Medicine, Changchun, China; ^3^Department of Cardiovascular Medicine, Affiliated Hospital of Changchun University of Chinese Medicine, Changchun, China; ^4^Center for Cancer Research, National Cancer Institute, Frederick, MD, United States

**Keywords:** pilose antler peptide, adriamycin, myocardial injury, protective effect, TGF-β/SMADs

## Abstract

Adriamycin (ADR)-based combination chemotherapy is the standard treatment for some patients with tumors in clinical, however, long-term application can cause dose-dependent cardiotoxicity. Pilose Antler, as a traditional Chinese medicine, first appeared in the Han Dynasty and has been used to treat heart disease for nearly a thousand years. Previous data revealed pilose antler polypeptide (PAP, 3.2KD) was one of its main active components with multiple biological activities for cardiomyopathy. PAP-3.2KD exerts protective effects againt myocardial fibrosis. The present study demonstrated the protective mechanism of PAP-3.2KD against Adriamycin (ADR)-induced myocardial injury through using animal model with ADR-induced myocardial injury. PAP-3.2KD markedly improved the weight increase and decreased the HW/BW index, heart rate, and ST height in ADR-induced groups. Additionally, PAP-3.2KD reversed histopathological changes (such as disordered muscle bundles, myocardial fibrosis and diffuse myocardial cellular edema) and scores of the heart tissue, ameliorated the myocardial fibrosis and collagen volume fraction through pathological examination, significantly increased the protein level of Bcl-2, and decreased the expression levels of Bax and caspase-3 in myocardial tissue by ELISA, compared to those in ADR-induced group. Furthermore, ADR stimulation induced the increased protein levels of TGF-β1 and SMAD2/3/4, the increased phosphorylation levels of SMAD2/3 and the reduced protein levels of SMAD7. The expression levels of protein above in ADR-induced group were remarkably reversed in PAP-3.2KD-treated groups. PAP-3.2KD ameliorated ADR-induced myocardial injury by regulating the TGF-β/SMAD signaling pathway. Thus, these results provide a strong rationale for the protective effects of PAP against ADR-induced myocardial injury, when ADR is used to treat cancer.

## Introduction

Adriamycin (ADR) is a broad-spectrum anthracycline antibiotic derived from *Streptomyces peucetius*. Clinically, ADR was used to treat acute leukemia, various malignant tumors, and other diseases (1). At therapeutic doses, it could cause a series of severe toxic reactions, including bone marrow suppression, nausea, vomiting, nephrotoxicity and cardiac toxicity (2). Such side effects were shown to limit its use and increase the incidence of cardiovascular disease and associated mortality in cancer survivors significantly (3, 4).

Pilose antler (Deer antler, *Cornu Cervi Pantotrichum* from *Cervusnippon Temminck*), a traditional Chinese medicine preparation, was mainly produced in Jilin, China. Pilose antler polypeptide (PAP), one main component of Pilose antler, has multiple biological activities, including the amelioration of inflammation, oxidative stress, organ injury, and fibrosis (5, 6). PAP exerted protective effects against myocardial fibrosis. However, its mechanism of action in myocardial injury was unclear (7). The transforming growth factor-β1 (TGF-β1)/Drosophila mothers against decapentaplegic proteins (SMADs) pathway had been considered to play an important role in the pathogeneses of myocardial infarction, cardiomyopathy and heart failure (8). When myocardial injury occurred, the overexpression of TGF-β1 induced the activation of SMADs and exacerbated disease progression (9). Since ADR toxicity in the heart of patients with cancer is observed while ADR is used to treat cancer, the protective roles and mechanisms of PAP against ADR-induced myocardial injury are investigated.

## Materials and Methods

### Reagents

ADR was obtained from Shanghai Aladdin Biochemical Technology Co., Ltd. (Shanghai, China). Hematoxylin and eosin (H&E) staining kit was purchased from Shanghai Beyotime Biotechnology Co., Ltd. (Shanghai, China). Enzyme-linked immunosorbent assay (ELISA) kits for cardiac troponin T (cTnT) and cardiac troponin I (cTnI) were purchased from Jiancheng Institute of Biotechnology (Nanjing, China). ELISA kits for B-cell lymphoma-2 (Bcl-2), Bcl-2-associated X protein (Bax), and caspase-3 were purchased from Boster Biological Technology Co., Ltd. (Wuhan, China). All antibodies were obtained from ProteinTech Group, Inc. (Wuhan, China).

### Extraction of Pilose Antler peptide

Pilose Antler (Deer antler, *Cornu Cervi Pantotrichum*, No. 20180325) obtained from Zhenyuan Deer Industry Co., Ltd., Jilin, China was confirmed. The voucher specimen was prepared and deposited at Department of Pharmacy in Changchun University of Chinese Medicine. Pilose antler polypeptide (PAP), one of its main active components, was isolated.

Fresh deer antler (1 kg) was chopped into 1 cm^3^ pieces at 4°C, the blood was quickly washed off with distilled water at 4°C, and then 1,000 ml of acetic acid solution (pH 4.0) was added, followed by colloid grinding, repeated homogenization, and centrifugation at 6,500 × g for 20 min at 4°C. The super solution was collected, 90% ethanol was added to a final concentration of 65%, and the mixture was stored at 4°C with stirring every 20 min for 6 h, followed by standing for 12 h. Subsequently, the mixture was centrifuged at 6,500 × g for 20 min at 4°C. The supernatant was collected and freeze-dried to obtain the crude extract of PAP, which was stored at −20°C. PAP was further separated by SuperdexG-75 gel chromatography column, and the components are collected according to the 280 nm ultraviolet absorption peak and then freeze-dried. Furthermore, Molecular weight (MW) of PAP was measured using western Blotting. MW of PAP are 3.2KD and 10KD separately. In the present study, 3.2 KD PAP (PAP-3.2KD, with a purity of 91%) was used for ADR-induced myocardial injury (Supplementary Figure 1) (10). The yield rate of PAP-3.2KD from fresh deer antler is 7.28%.

### Animals

Forty 6-week-old Wistar rats (male to female ratio = 1:1, weighing 200–240 g) were obtained from Changchun Yisi Experimental Animal Technology Co., Ltd. (Changchun, China). They were maintained under standard conditions (23°C, constant relative humidity as 26% and 12-h dark/light cycle). All experimental procedures for the care and use of laboratory animals and animal handling followed the guidelines of the National Animal Welfare Law of China. The protocols (No. 20180003) for the animal experiments were approved by the Ethics Committee of Changchun University of Chinese Medicine.

### Experimental Groups and Treatments

The rats were randomly divided into the following four groups: control, ADR and ADR+PAP-3.2KD 100 and 200 mg/kg, 10 rats in each group. Except the control group, Rats from the other three groups were intraperitoneally injected with ADR (2.5 mg/kg) every 2 days for six times at a cumulative dose of 15 mg/kg (11–13). The rats in control group were intraperitoneally injected with an equal volume of normal saline (ADR solution medium was saline, PAP-3.2KD or ADR solution medium had no effect on healthy rats, data not shown). The rats from two PAP-3.2KD groups were separately administrated orally with PAP-3.2KD 100 or 200 mg/kg for 21 days after six-time ADR stimulation. The rats from ADR group were given orally with an equal volume of water. After 21-days PAP-3.2KD or water treatment, the rats were starved for 12 h, and were anesthetized by an intraperitoneal injection of 3% sodium pentobarbital (35 mg/kg). Blood samples (6–10 mL) were collected from the aorta abdominalis and centrifuged at 1,000 × g for 10 min at 4°C. The serum samples were then stored at −80°C for future analysis. The cardiac tissues of rats were harvested.

### Body Weight, Heart Weight, and HW/BW Index Assay

The rats in each group were weighed every 3 days. After the rats were euthanized, the hearts were isolated and weighed to calculate the HW/BW index.

### Heart Rate and ST Height Measurement

According to the animal experimental ethics inspection form of Changchun university of Chinese medicine, all rats were anesthetized with isoflurane and fixed in a supine position 30 min following the final drug administration. The Powerlab biological signal acquisition and processing system (AD Instruments, Sydney, Australia) were connected to an electrocardiogram to record heart rate and ST height within 2 min.

### Myocardial Histopathology

Heart tissue specimens were fixed in normal 4% paraformaldehyde for 48 h and dehydrated using a graded series of alcohol concentrations. After the specimens had been embedded and sliced, they were stained with H&E (magnification, ×200; Olympus, Tokyo, Japan). The severity of pathological changes was evaluated and graded by two independent observers according to the evaluation criteria shown in Supplementary Table 1.

### Masson's Trichrome Stain

Myocardial fibrosis was detected by Masson's trichrome staining. Frozen tissue sections were fixed in Bouin's solution for 1 h at 56°C, followed by staining according to the manufacturer's protocol from Trichrome Stain (Masson) kit (Solable Technology, Beijing, China). Under light microscopy, collagen fibers were stained for blue, while cardiomyocytes were stained for red. The collagen area in each field was measured using Image-Pro Plus 6.0 image analysis software, and the collagen volume fraction (CVF) was calculated as follows: CVF = (collagen area/myocardial area) ×100%.

### TUNEL

The heart tissues of rats were cut into 5 μm paraffin sections. Myocardial apoptosis was detected by TUNEL staining, according to the commercial kit protocols (Beyotime Biotechnology Co. Ltd., Shanghai, China). TUNEL mix contained 50 μL enzymesolution and 450 μL label solution. Heart sections were incubated with 50 μL TUNEL mix at 37°C for 1 h. The sections were washed in PBS and stained with DAB for 30 min. Methyl green complex staining was carried out. After PBS wash, the sections were mounted and observed. Under light microscopy, the normal myocardial cell nucleus was blue-green, and the apoptotic cells were dark brown in different shades. Each slice was randomly selected with 5 high-power fields (×400). The percentage of myocardial apoptosis area to myocardial area was referred to as the apoptosis index (AI).

### ELISA

Serum cTnT and cTnI concentrations were determined to evaluate myocardial injury, according to the commercial kit protocols (Jiancheng Institute of Biotechnology, Nanjing, China). The serum samples were added to the wells pre-coated with antibody in one plate, and were incubated with HRP labeled first antibody. Plates were analyzed with a spectrophotometer after incubation with substrate (450 nm).

Myocardial tissues were homogenized at 4°C with lysis buffer (PBS containing 0.05% sodium azide, 0.5% Triton X-100, and a protease inhibitor cocktail, pH 7.2) (Jiancheng Institute of Biotechnology, Nanjing, China). The expression levels of Bcl-2, Bax, and caspase-3 were determined according to the ELISA kit protocols (Boster Biological Technology, Co. Ltd.).

### Western Blotting

Total protein in the heart tissues was extracted according to the instructions of the protein extraction kits (Solaibao Technology, Co. Ltd., Beijing, China), and quantified using bicinchoninic acid (BCA) protein assay kits (SolarBio, Beijing, China). Equal amounts of protein were loaded and separated using 8–12% sodium sulfate dodecyl-polyacrylamide gel electrophoresis (SDS-PAGE) and then transferred onto PVDF membranes, which were blocked in 5% skimmed milk at 25°C for 1 h on a shaking table. Membranes were incubated with the appropriate concentrations of the following antibodies (Proteintech Group, Inc., Wuhan, China) at 4°C overnight: anti-Bcl-2 (1:2,000, Cat. No. 12789), anti-Bax (1:4,000, Cat. No. 50599), anti-Caspase-3 (1:2,000, Cat. No. 19677), anti-TGF-β (1:1,000, Cat. No. 21898, anti-SMAD2 (1:6,000, Cat. No. 12570), anti-SMAD3 (1:1,000, Cat. No. 25494), anti-SMAD4 (1:1,000, Cat. No. 10231), anti-SMAD7 (1:2,000, Cat. No. 25840), and anti-glyceraldehyde 3-phosphate dehydrogenase (GAPDH, 1:40,000, Cat. No. 10494). After three rounds of washing with Tris-buffered saline plus Tween (TBST), the membranes were incubated with a secondary anti-rabbit antibody (1:10,000, cat. no. 21991) at room temperature (RT) for 1 h. Finally, the immunoreactive bands were visualized using an enhanced chemiluminescence kit with a gel imaging system (Proteintech Group, Inc.).

### Statistical Analysis

The results were presented as the means ± standard error of mean (SEM). Differences between groups were analyzed using one-way analysis of variance (ANOVA) with Tukey's multiple comparison test. A *P* < 0.05 was considered significant, and all data analyses were conducted using the statistical package for the social sciences (SPSS) 21.0 software (SPSS Inc., Chicago, IL, USA).

## Results

### PAP-3.2KD Reversed HW/BW Index in ADR-Induced Rats

**Figure 1:**
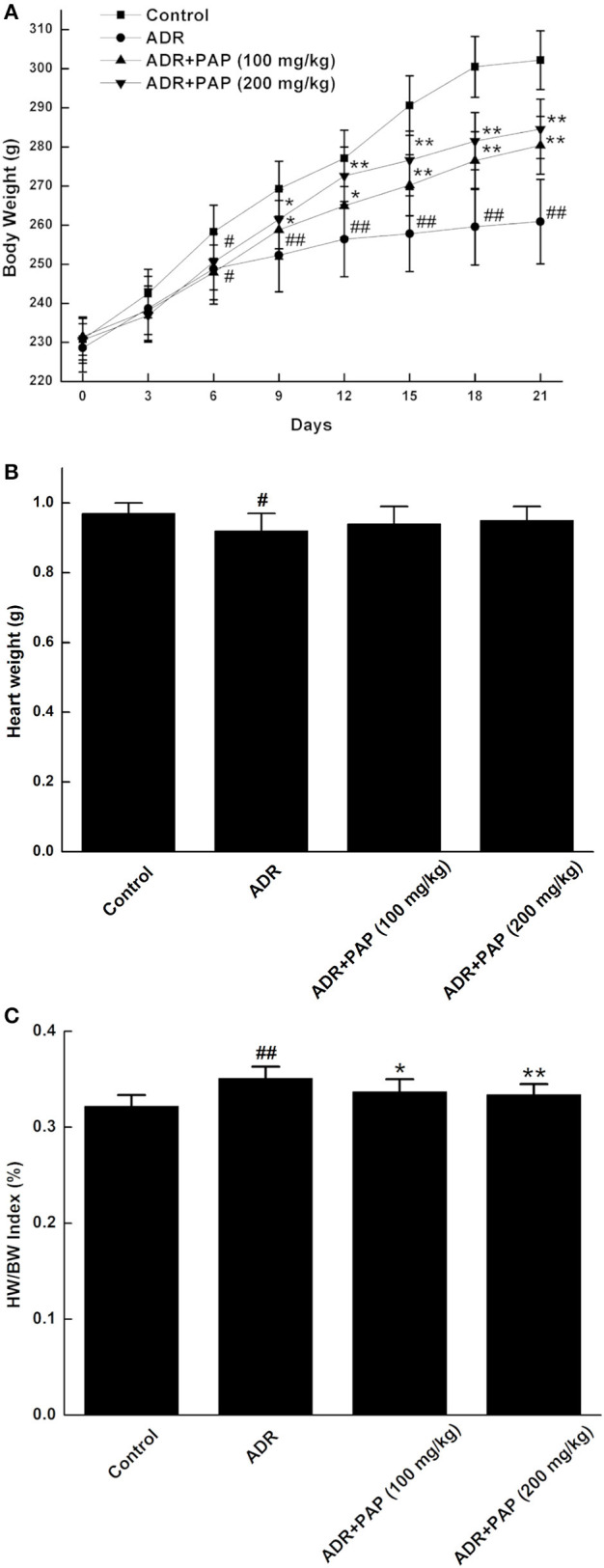
PAP-3.2KD reversed HW/BW index of ADR-induced rats. **(A)** Every 3 days, body weight (BW) was measured in four groups, includingcontrol, ADR, ADR+ PAP-3.2KD (100 mg/kg), ADR+ PAP-3.2KD (200 mg/kg). **(B,C)** Both Heart weight (HW) and HW/BW index were detected in four groups after the rats were euthanized. Data were presented as Mean ± standard error of mean (*n* = 10). ^#^*P* < 0.05, ^##^*P* < 0.01 vs. the control group; *P < 0.05, **P < 0.01 vs. the ADR group, ADR, Adriamycin; PAP-3.2KD, 3.2 KD pilose antler polypeptide.

After 6 days of PAP-3.2KD treatment, three experimental groups including ADR and PAP-3.2KD (100 and 200 mg/kg) treatment displayed significantly lower BW than those in the control group. BW levels in two PAP-3.2KD groups (100 and 200 mg/kg) groups were significantly increased at 12 days after PAP-3.2KD administration, compared to those in the ADR group (*P* < 0.05 and <0.01), even though BW levels in two PAP-3.2KD groups were still significantly lower than those in the control group (Figure 1A).

The heart weights of rats in the ADR group were significantly reduced compared with those in the control group (*P* < 0.05). After treatment with PAP-3.2KD, the heart weights were increased, even though there was no significant difference, compared to those from ADR group (*P* > 0.05; Figure 1B). In addition, The HW/BW index was calculated, since it is a basic indicator of cardiac edema and hyperplasia, which reflects the degree of myocardial injury. HW/BW index of rats in the ADR group was increased compared to that in the control rats (*P* < 0.01). The HW/BW index of rats in two PAP-3.2KD groups (100 and 200 mg/kg) were markedly decreased, compared to those in ADR groups separately (*P* < 0.05 and *P* < 0.01; Figure 1C).

### PAP-3.2KD Recovered Heart Rate and ST Height in ADR-Induced Rats

**Figure 2:**
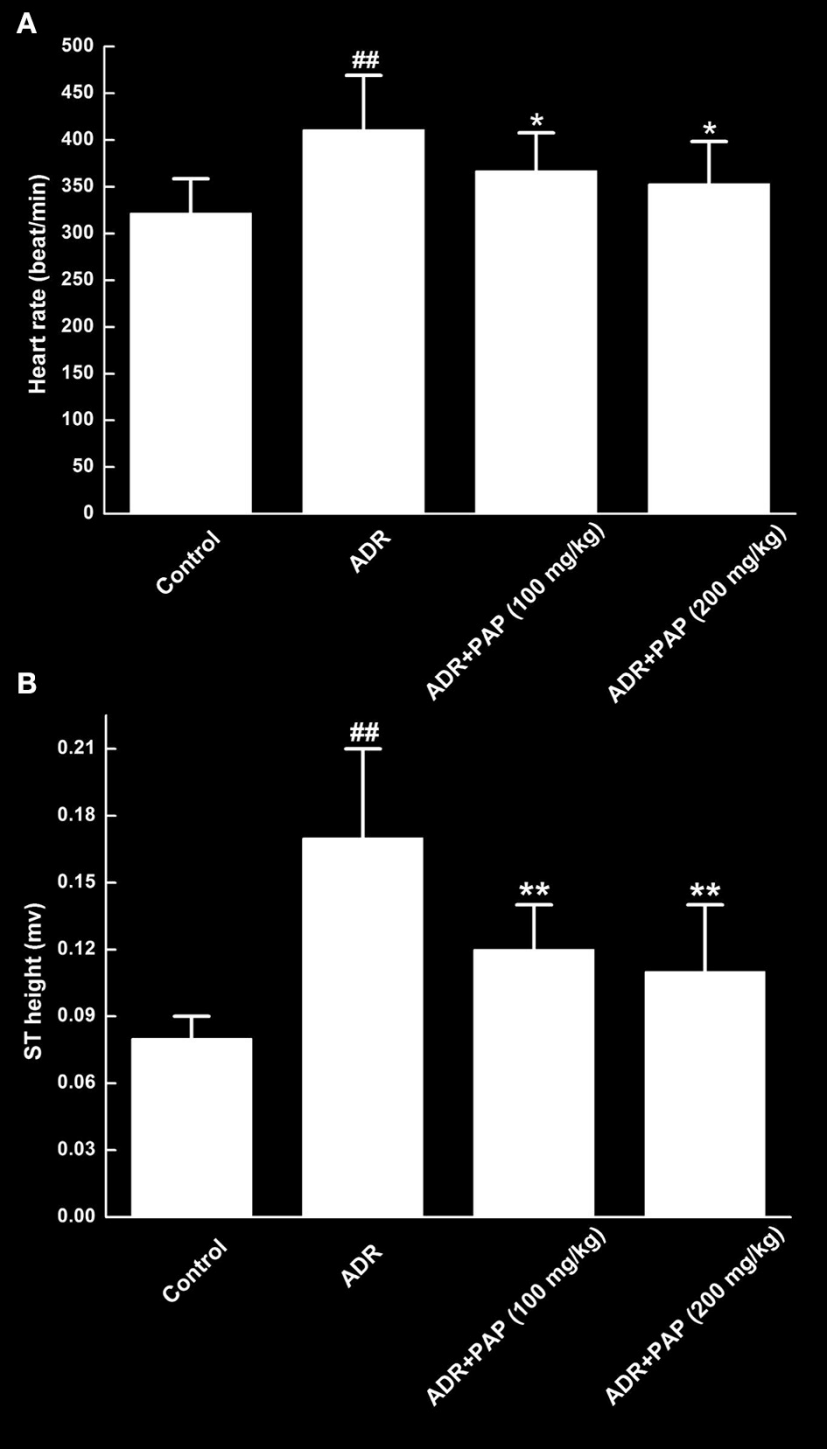
PAP-3.2KD recovered heart rates and ST heights of ADR-induced rats. Both heart rate **(A)** and ST height **(B)** were determined by an eight-channel biological signal acquisition and processing system. Data were presented as Mean ± standard error of mean (*n* = 10). ^##^*P* < 0.01 vs. the control group; *P < 0.05, **P < 0.01 vs. the ADR group.

To study the effects of PAP-3.2KD and/or ADR on cardiac function, heart rates and ST heights of ADR-induced rats were measured. Both heart rates and ST heights were remarkably increased by ADR induction compared to those of the control group (*P* < 0.01). However, in the two PAP-3.2KD treatment groups, there were the obvious decrease in these parameters compared to those in the ADR group separately (*P* < 0.05 and <0.01), close to those in control group (Figure 2).

### Protective Roles of PAP-3.2KD on Serum cTnT and cTnI

**Figure 3:**
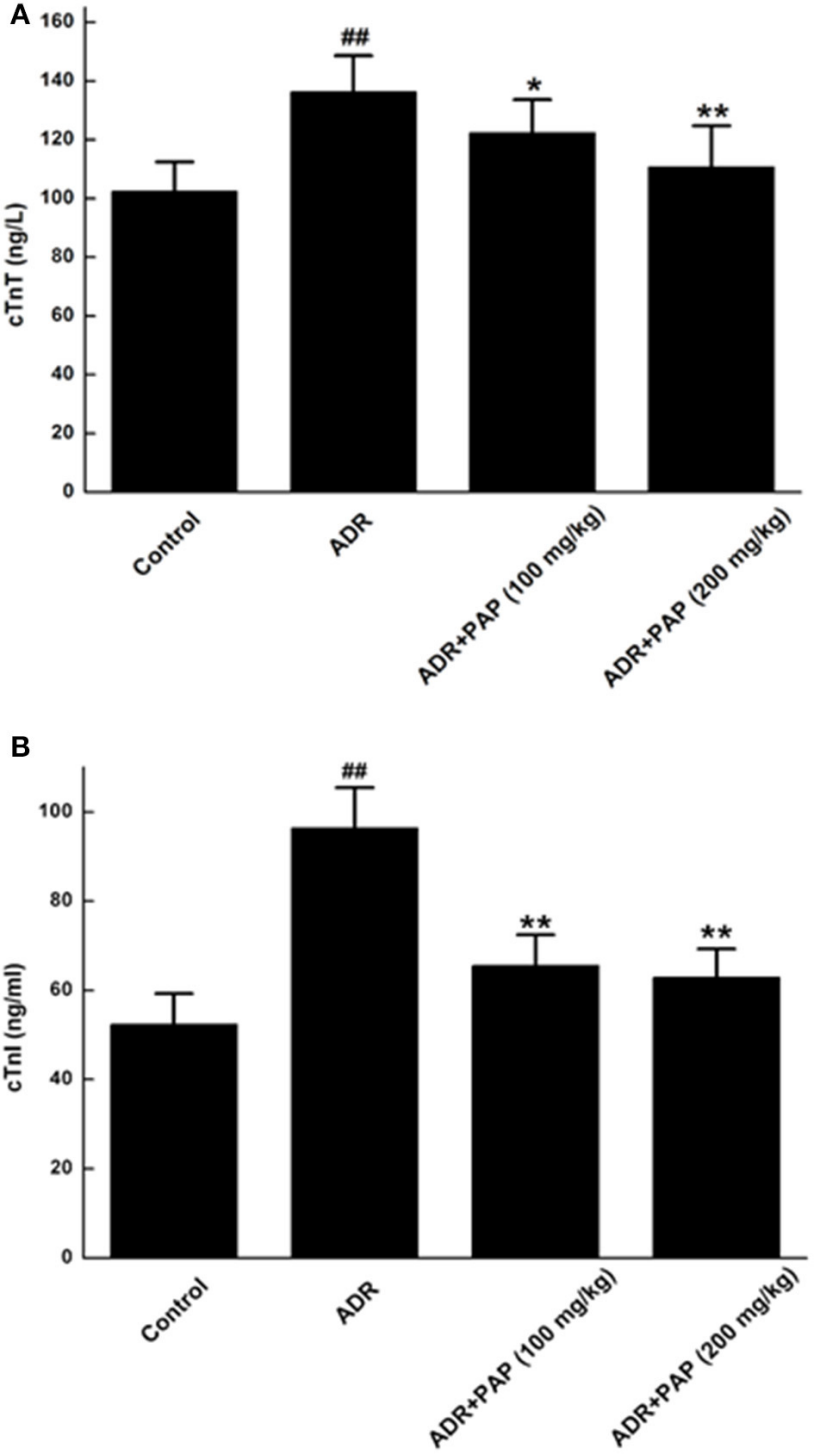
PAP-3.2KD restored the levels of cTnT and cTnI in ADR-induced rats. Serum concentrations of both cTnT **(A)** and cTnI **(B)** were determined by ELISA. Data were presented as Mean ± standard error of mean (*n* = 10). ^##^*P* < 0.01 vs. the control group; *P < 0.05, **P < 0.01 vs. the ADR group, Cardiac troponin T (cTnT); Cardiac troponin I (cTnI).

The levels of serum cardiac troponin T (cTnT) and cardiac troponin I (cTnI) in ADR group were increased significantly compared to those of the control group. After PAP-3.2KD (100 and 200 mg/kg) treatment, the levels of cTnT and cTnI were markedly decreased relative to those of the ADR group (Figure 3).

### PAP-3.2KD Attenuated the Histopathological Damages of ADR-Induced Myocardial Tissue

**Figure 4:**
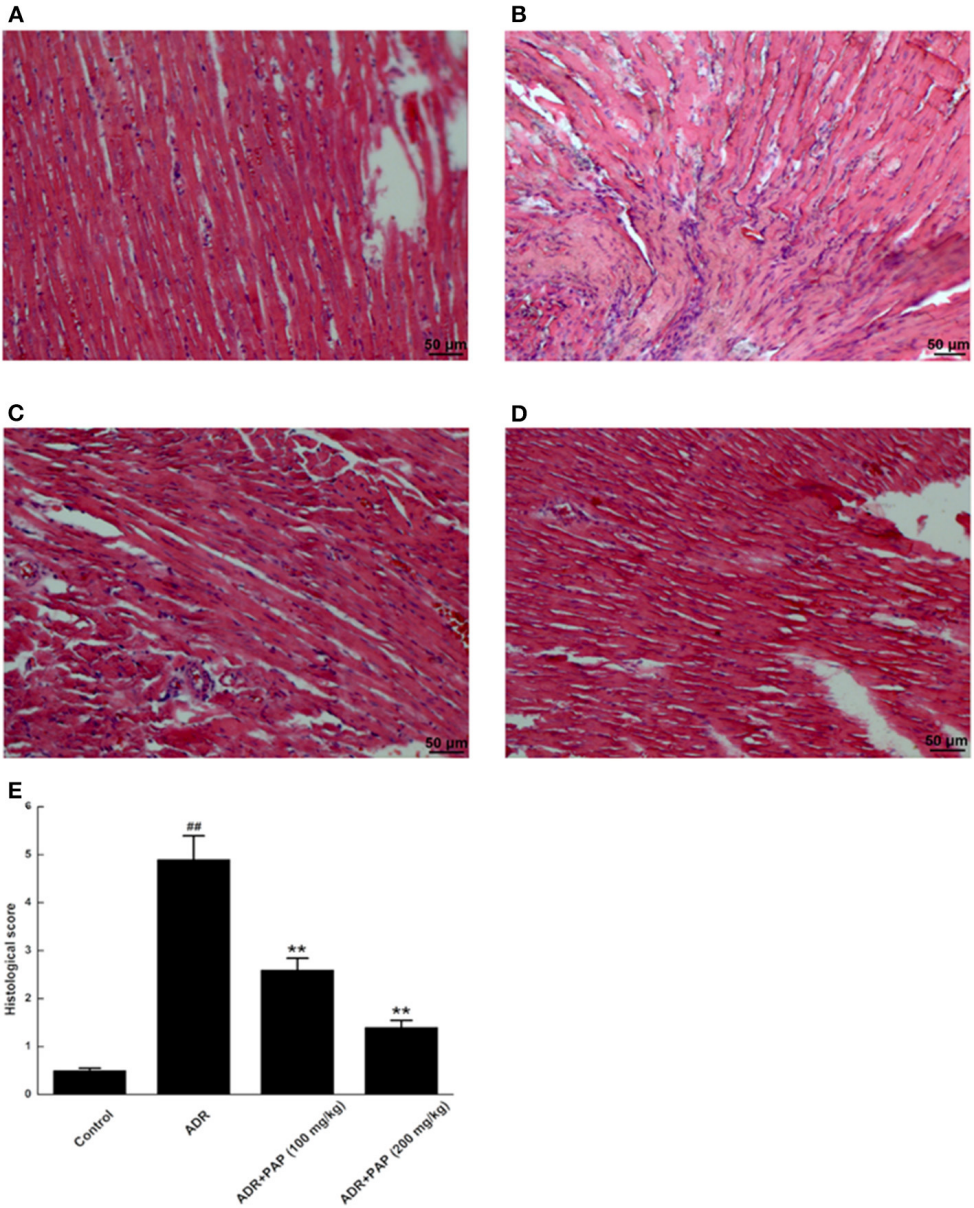
PAP-3.2KD ameliorated the histopathological damages of ADR-induced myocardial tissue. Histopathological damages of myocardial tissues were detected by HE staining. **(A)** Control group, **(B)** ADR group, **(C)** PAP-3.2KD group (100 mg/kg), **(D)** PAP-3.2KD group (200 mg/kg). (Magnification, ×200). **(E)** Mean histopathological scores in each group. Data were presented as Mean ± standard error of mean (*n* = 6). ^##^*P* < 0.01 vs. the control group; **P < 0.01 vs. the ADR group.

In the control group, the pathological changes of heart tissues were rarely observed, and well-organized muscle bundles and no broken muscle fibers were found (Figure 4A). For the heart tissues of the ADR group, the typical characteristics of myocardial injury was found, such as disordered muscle bundles, myocardial fibrosis and diffuse myocardial cellular edema (Figure 4B). In the PAP-3.2KD groups, relatively well-organized muscle bundles, lower levels of myocardial fibrosis and diffuse myocardial cellular edema were revealed compared to those in the ADR-stimulated group (Figures 4C,D). To evaluate the damage of myocardia tissues quantitatively, the pathological scores were measured based on the ratios of pathological change areas (including inflammation, myocardial fibrosis and diffuse myocardial cellular edema) to the areas of the viewed entire field, as described in Supplementary Table 1. The pathological scores from the ADR group were significantly higher than those from the control group, whereas the scores in the PAP-3.2KD (100 and 200 mg/kg) groups were significantly attenuated, compared to those in ADR group separately (*P* < 0.01, both; Figure 4E). Those results indicated that PAP-3.2KD alleviated ADR-induced pathological injury of the myocardial tissues.

### PAP-3.2KD Ameliorated ADR-Induced Myocardial Fibrosis

**Figure 5:**
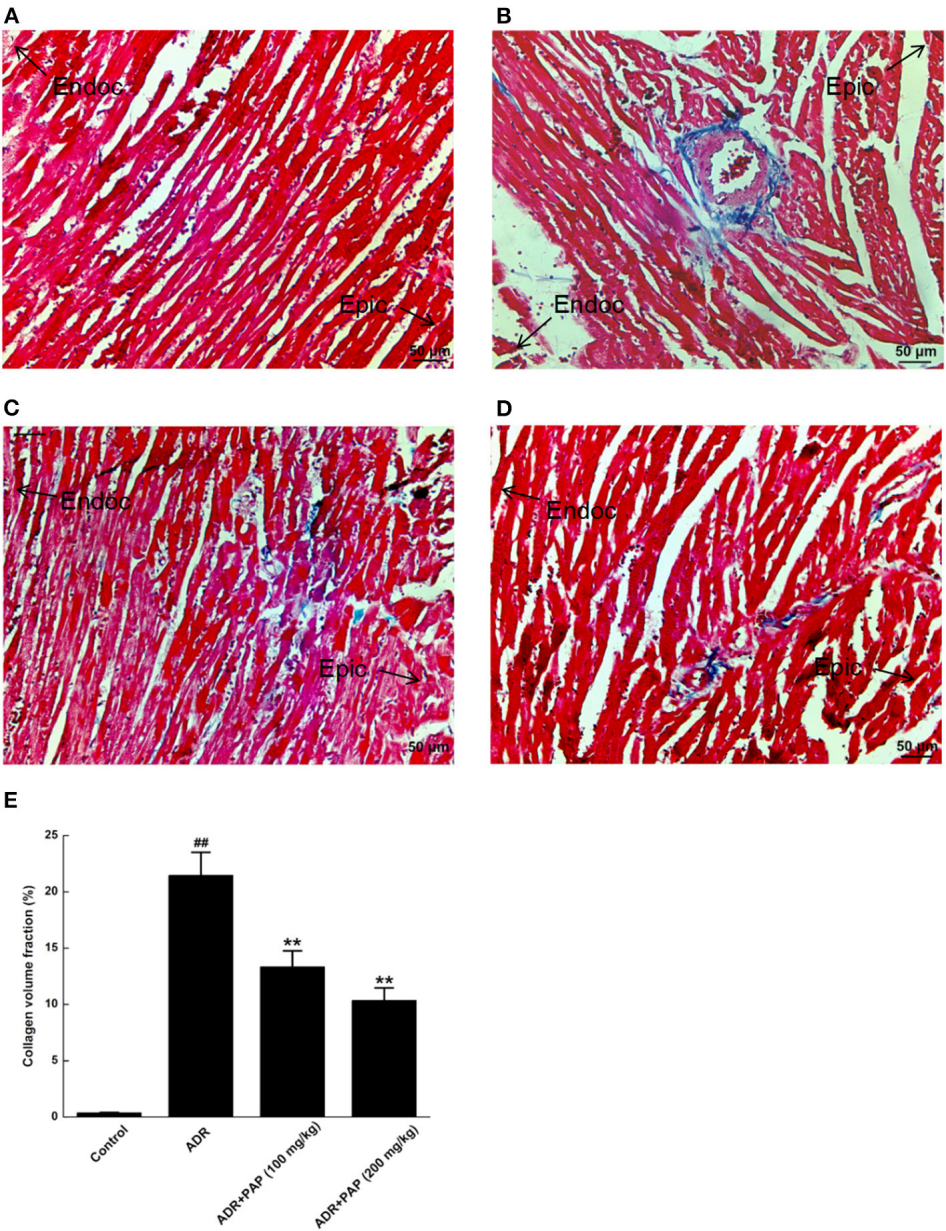
PAP-3.2KD inhibited ADR-induced myocardial fibrosis. Myocardial Fibrosis in each group were detected by Masson's trichrome staining. **(A)** Control group, **(B)** ADR group, **(C)** PAP-3.2KD group (100 mg/kg), **(D)** PAP-3.2KD group (200 mg/kg). (Magnification, ×200). **(E)** Collagen volume fraction (CVF) was calculated in each group. All the myocardial section were made by Left ventricular apex. The zone of endocardium (Endoc)-epicardium (Epic) was indicated. Data were presented as Mean ± standard error of mean (*n* = 6). ^##^*P* < 0.01 vs. the control group; **P < 0.01 vs. the ADR group.

The fibrotic areas were stained blue, after Masson's trichrome staining. There was no obvious fibrosis in the myocardium of the control group (Figure 5A). Compared with the control group, fibrosis in myocardial tissue from the ADR group was increased significantly (Figure 5B). Histological quantification results were demonstrated with the collagen volume fraction (CVF). After PAP-3.2KD administration, the CVF was decreased with the reduction of fibrosis significantly (Figures 5C–E). Thus, PAP-3.2KD reduced ADR-induced cardiac fibrosis.

### PAP-3.2KD Reduced the Apoptosis of ADR-Induced Myocardial Tissue

**Figure 6:**
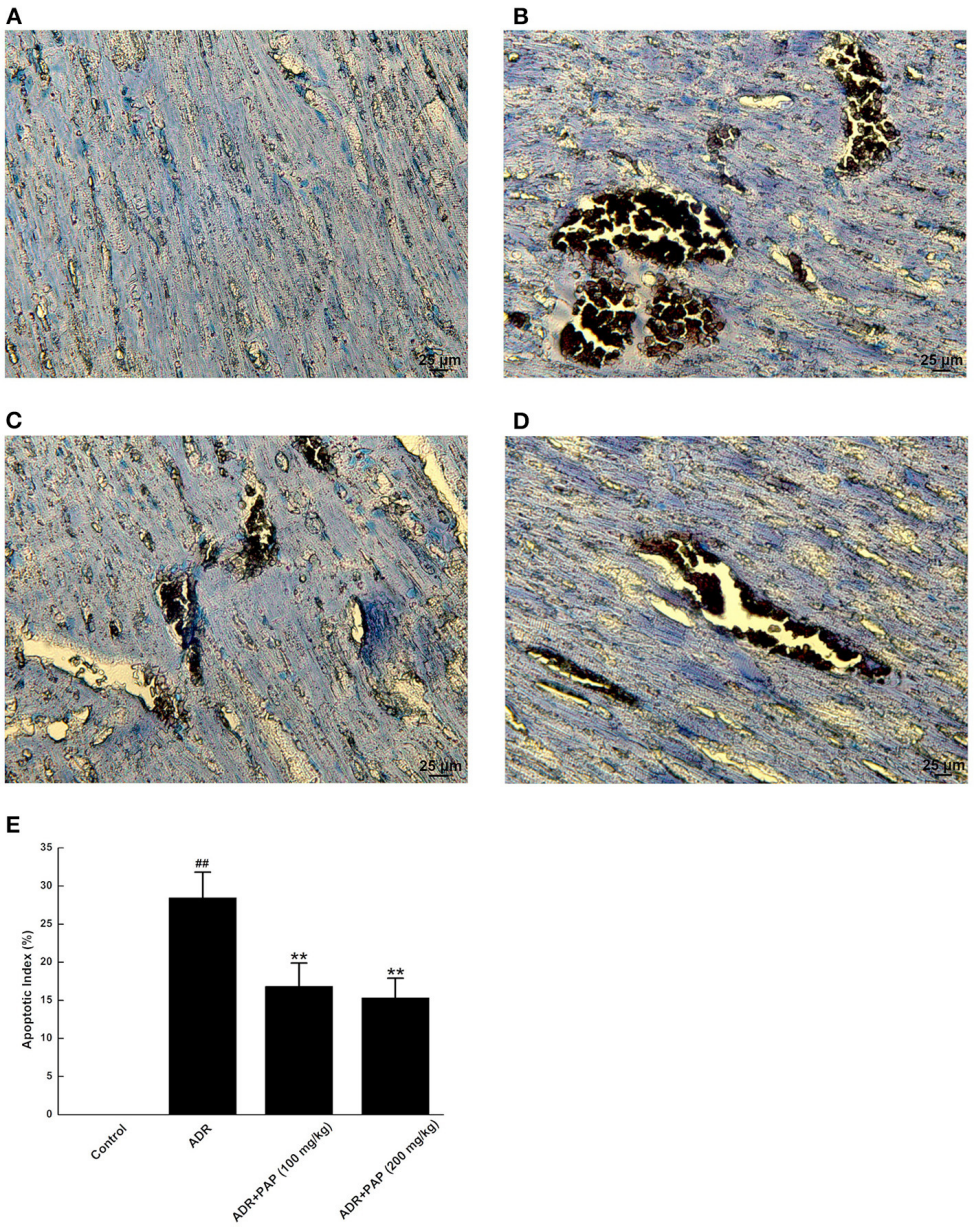
PAP-3.2KD blocked the apoptosis of ADR-induced myocardial tissue. Apoptosis of myocardial tissues was detected by TUNEL staining. TUNEL-positive cells exhibited dark buffy nuclei staining. **(A)** Control group, **(B)** ADR group, **(C)** PAP-3.2KD group (100 mg/kg), **(D)** PAP-3.2KD group (200 mg/kg). (Magnification, ×400). **(E)** Statistical analysis of apoptotic index in each group. The independent experiments (*n* = 6) were performed in each group. ^##^*P* < 0.01 vs. the control group; **P < 0.01 vs. the ADR group.

TUNEL staining results demonstrated that there was no obvious apoptosis in the myocardium of the control group. The nucleus of normal cardio myocytes was blue-green, the apoptotic cells were rarely found for dark brown in different shades, the muscle bundles were neatly arranged and the cell gap was uniform (Figure 6A). In ADR group, myocardial tissue apoptosis was obvious. The dark brown areas with different sizes appeared and the arrangement of myocardial muscle bundles was relatively disordered (Figure 6B). The muscle bundles in both PAP-3.2KD group were relatively neatly arranged, and dark brown areas were reduced, especially in the PAP-3.2KD (200 mg/kg) group (Figures 6C,D). The apoptosis areas in myocardial tissue were decreased significantly after PAP-3.2KD treatment (*P* < 0.01; Figure 6E).

**Figure 7:**
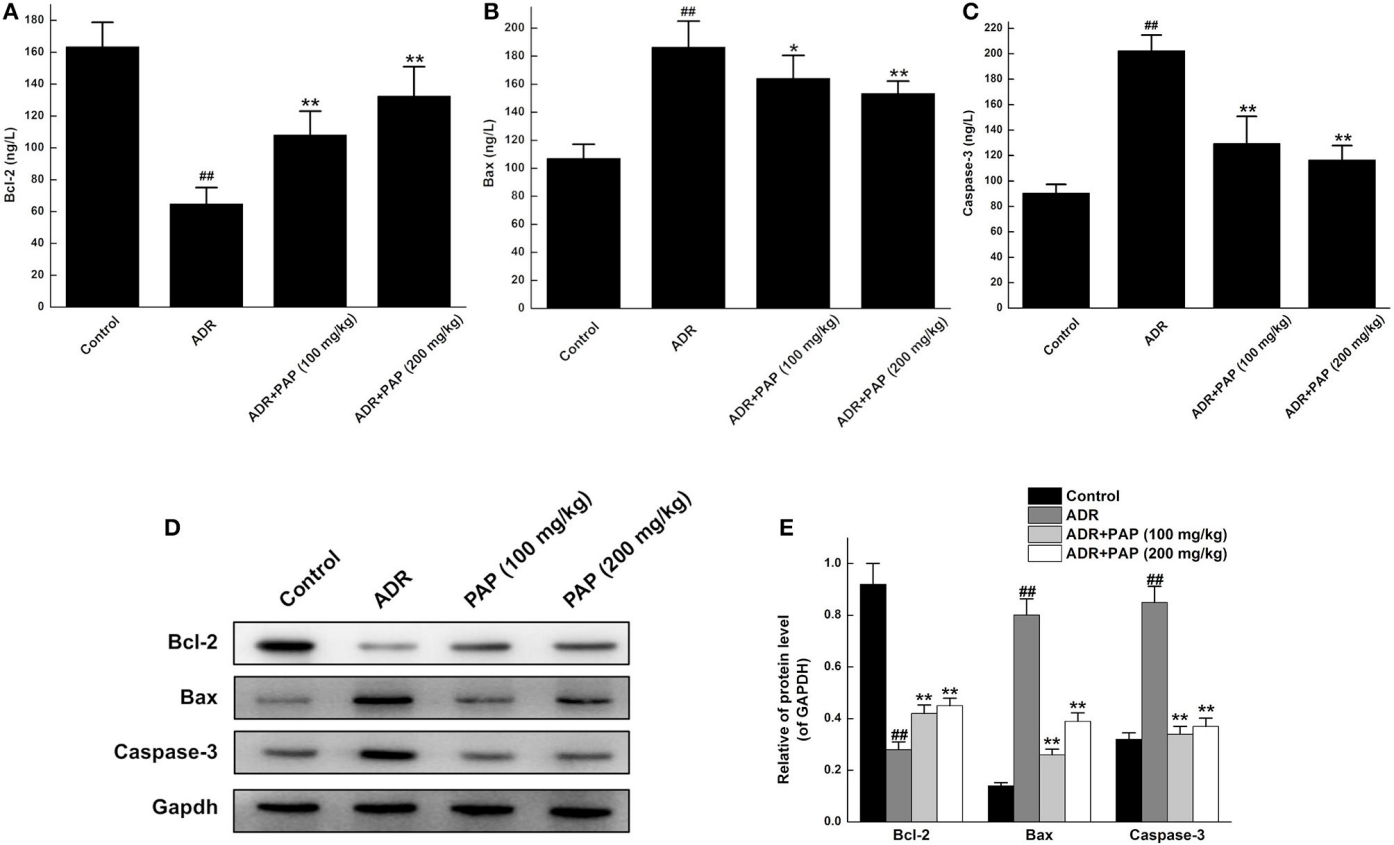
PAP-3.2KD regulated the expression levels of Bcl-2, Bax, and caspase-3 in ADR-induced rats. The concentrations of Bcl-2 **(A)**, Bax **(B)** and Caspase-3 **(C)** in cardiac tissue were determined by ELISA. Data were presented as Mean ± standard error of mean (*n* = 10). **(D)** The protein expression levels of Bcl-2, Bax and Caspase-3 were determined by western blotting. **(E)** Statistical analysis for expression levels of relative protein in **(D)**, GAPDH protein levels as control. Data were presented as Mean ± standard error of mean (*n* = 3). ^##^*P* < 0.01 vs. the control group; *P < 0.05, **P < 0.01 vs. the ADR group, ADR, Adriamycin; PAP-3.2KD, 3.2 KD pilose antler polypeptide; Bcl-2, B-cell lymphoma-2; Bax, BcL-2-Associated X.

The functional pathways of PAP-3.2KD on ADR-induced apoptosis of cardiac myocytes were examined further. The expression levels of apoptotic protein, Bcl-2, Bax, and caspase-3 in cardiac tissue were analyzed with ELISA and Western blotting. Bcl-2 expression level in myocardial tissue was decreased with the increased expression of Bax and caspase-3 in the ADR group significantly, compared to the expression levels of those protein in the control group (All, *P* < 0.01). However, after PAP-3.2KD treatment, the expression levels of those proteins in the ADR group were reversed. Compared with expression levels of those proteins in the ADR group separately, Bcl-2 expression levels in both PAP-3.2KD (100 and 200 mg/kg) group were significantly increased with the distinctly reduced expression levels of Bax and caspase-3 (*P* < 0.05 and <0.01; Figure 7).

### PAP-3.2KD Attenuated ADR-Induced Myocardial Injury Through TGF-β_1_/SMAD Pathway

**Figure 8:**
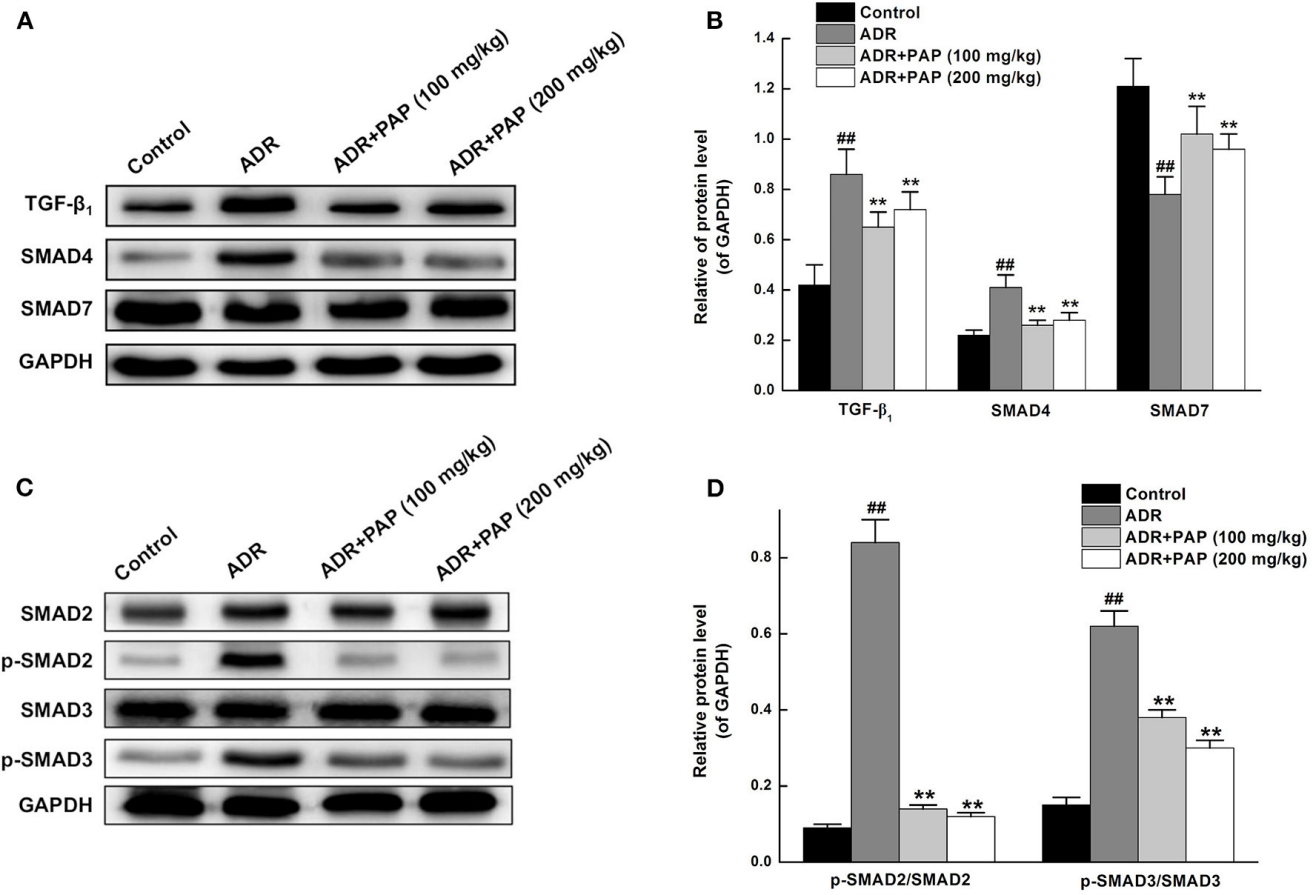
PAP-3.2KD attenuated ACR-induced myocardial injury through TGF-β/SMAD pathway. **(A)** The protein expression levels of TGF-β_1_, SMAD4, and SMAD7 in heart tissues were determined by western blotting. **(B)** Statistical analysis of relative levels of target protein in **(A)**, compared to GAPDH protein levels. The representation **(C)** and quantitative analysis **(D)** of western analysis results for SMAD2/SMAD3 and their phosphorylation. Coenzyme Q10 (CoQ10) as positive control group (CoQ10 has been proved to have protective roles for cardiomyocyte after myocardial injury through TGF-β pathway, Data not shown). Thus, splicing occurred between ADR group (Lane 2) and PAP-3.2KD (100 mg/kg) (Lane 3) group in all Western Blotting results. Data were expressed as mean ± standard error of mean (*n* = 3). ^##^*P* < 0.01 vs. the control group; **P < 0.01 vs. the ADR group. TGF-β_1_, transforming growth factor-β_1_; SMAD, drosophila mothers against the decapentaplegic protein.

The treatment mechanism of PAP-3.2KD on ADR-induced myocardial injury was investigated further. The expression levels of TGF-β1, a major profibrotic cytokine in hearts, were first measured using Western blot analysis. The result demonstrated that PAP-3.2KD treatment inhibited ADR-induced TGF-β1 production. In addition, the increased expression of TGF-β-related SMAD4 and reduced expression of SMAD7 in ADR-induced groups were also efficiently reversed after PAP-3.2KD treatment (100 and 200 mg/kg) (All, *P* < 0.01; Figures 8A,B). Compared with control group, there were no significant changes in total protein levels of SMAD2/SMAD3 from ADR group, whereas, there were significantly increased phosphorylation levels of them. PAP-3.2KD (100 and 200 mg/kg) treatment obviously reversed the phosphorylation levels of SAMD2/SMAD3, which were induced by ADR (All, *P* < 0.01; Figures 8C,D). These results indicate that PAP-3.2KD treatment may attenuate ADR-induced myocardial injury through the TGF-β_1_/SMAD pathway.

## Discussion

In the present study, there were two key novel findings, the first was that PAP-3.2KD reversed ADR-induced pathological changes such as abnormal HW/BW index/heart rate/ST height, damage of the heart tissue, myocardial fibrosis and collagen volume fraction. PAP-3.2KD also increased the protein level of Bcl-2, and decreased the expression levels of Bax and caspase-3 in myocardial tissue, compared to those in ADR-induced group. The second was that PAP-3.2KD treatment blocked ADR-induced apoptosis and inflammatory response. ADR was effective when combined with chemotherapy for the treatment of various tumors; however, its toxic side effects caused multiple organ damage (14). ADR has specific toxicity toward the heart due to the high binding affinity of ADR to the anionic phospholipid cardiolipin within the inner mitochondrial membrane of the myocardial cells (15). The clinical application of ADR was restricted due to its side effect on cardiac tissues. Therefore, many studies have been focused on the protective effects of antioxidants and natural products against ADR-induced myocardial injury. In the present study, the HW/BW index was first checked as the most rational indication of changes in heart tissue pathology and under normal conditions, their levels were relatively constant (16). ADR induction significantly increased the HW/BW index, since BW levels were reduced more than those of HW (Figure 1), and impaired cardiac function through increasing the levels of heart rate and ST (Figure 2) and protein levels of cTnT/cTnI (Figure 3). Those results above were consistent with those from previous reports about ADR-induced abnormalities of cardiac function (17, 18). In the present study, PAP-3.2KD treatment significantly ameliorated the increased HW/BW index and the increased levels of heart rate/ST and cTnT/cTnI in ADR-treated rats, confirming its cardio-protection property (Figures 1–3). Even though the results showed no different between male and female rats, sex different effect still need to be studied further.

The two major mechanisms underlying these effects may be involved. First, the abnormal myocardial functions may be caused by notable pathological changes in the myocardial cells, such as disordered muscle bundles and myocardial fibrosis, which were found to be induced by ADR. Cardiac fibrosis is a process of pathological extracellular matrix (ECM) remodeling, leading to excessive and continuous ECM deposition in heart, and impairing heart muscle function above. PAP-3.2KD enhanced cardiac function through reducing the fibrosis and inflammation which ADR stimulated in myocardial tissues significantly (Figures 4, 5) (PAP-3.2KD was revealed to have no toxic effects in myocardial tissues from healthy rats, data not shown). Cardiomyocyte apoptosis was another mechanism by which ADR induced cardiomyopathy (Figure 6). ADR induced cardiac oxidative stress to disturb mitochondrial membrane permeability, resulting in the released cytochrome c into the cytosol. The cytochrome c bound to another protein to activate caspase cascade and induce cell death (19). Caspase-3 played a key role in apoptosis, especially in the core of the apoptosis cascade. Caspase-3 effectively hydrolyzed cellular structural and functional proteins, inducing cell apoptosis in different pathological status, such as myocardial tissues during myocardial injury (20, 21) (Figure 7). Bcl-2 and Bax were a pair of genes that regulated cell apoptosis to activate the next level caspases enzyme system to cause cell apoptosis (22). ADR induced cardiomyocyte apoptosis through the decreased expression level of Bcl-2 and the increased expression of Bax and caspase-3 (Figure 7). ADR-treated rats exhibited more myocardial injuries than those from control group. The phenomenon was also confirmed by the electrocardiogram experiment. The reduction in myocardial fibrosis and cardiomyocyte apoptosis may be an effective strategy to reduce the incidence of ADR-associated myocardial injuries. Compared with the levels of Bcl-2, caspase-3, and Bax in the ADR group, PAP-3.2KD significantly reversed their levels, inhibiting the occurrence of myocardial apoptosis (Figures 6, 7). Therefore, PAP-3.2KD administration reversed these symptoms to reduce ADR-induced myocardial injuries.

The expression levels of TGF-β1 protein were increased in cardiac tissue after ADR induction. After PAP-3.2KD administration on ADR-induced rats, TGF-β1 expression was effectively inhibited, protecting the myocardium from damage, suggesting that both ADR induced myocardial injury and PAP-3.2KD protected ADR-induced injury through TGF-β1 signaling (Figure 8). This was consistent with previous data that ADR promoted fibrosis and apoptosis by upregulation of TGF-β1 (23, 24). TGF-β1, as one multifunctional cytokine, regulated a number of biological responses including fibro-genesis and cell apoptosis, especially on various pathophysiological functions of the cardiovascular system (25–27).

The primary TGF-β signal transduction pathway is the highly conserved TGF-β/SMAD pathway. SMAD proteins, as the main intracellular signal transduction, mediated the transport of TGF-β signals from the cell membrane receptors to the nucleus. SMADs are the target proteins of TGF-β1 signal transduction, which transmits signals in the cell mainly through phosphorylation, such as SMAD2/3, which in turn bind to the common SMAD4 (28). SMAD7 binds to activated type I receptors to regulate signal transduction by the TGF-β family (29). The SMAD complex translocate to the nucleus, where they bind to DNA elements to promote the transcription of various genes that regulate fibrosis. ADR upregulated TGF-β1 expression to promote fibrosis which was a reparative response by which the myocardium compensated for cell loss after myocardial injury (30). PAP-3.2KD treatment inhibited the production of TGF-β_1_ and phosphorylation of SMAD2/3 induced by ADR. Therefore, PAP-3.2KD reduced myocardial fibrosis through TGF-β/SMAD signaling. Once the myocardium was damaged, TGF-β1 was activated and secreted, inducing increased apoptosis in cardiac cells (31, 32). ADR-induced myocardial injury down-mediated the levels of TGF-β1 to upregulate the protein expression of p-SMAD2/3, ultimately promoting ADR-induced apoptosis of myocardial cells, and PAP-3.2KD treatment on ADR-induced rats inhibited myocardial apoptosis through the inhibition of TGF-β/SMAD pathway (Figure 8). Even though the protective mechanism of PAP-3.2KD against ADR-induced myocardial injury may be clarified, other mechanisms, by which ADR-induced myocardial apoptosis need be investigated further.

## Conclusion

PAP-3.2KD treatment protects ADR-induced pathological changes, and inhibits ADR-induced myocardial fibrosis and myocardial apoptosis through the inhibition of TGF-β/SMAD pathway, suggesting that PAP-3.2KD may be one potential protective drug during cancer treatment of ADR.

## Data Availability Statement

All datasets generated in present study are included in the article/Supplementary Material.

## Ethics Statement

The animal study was reviewed and approved by Department of Pharmacy Changchun University of Chinese Medicine.

## Author Contributions

XQ conceived and designed the work. XQ and XH coordinated technical support and funding. YX and PQ wrote the manuscript. JZ, GL, and YL performed the experiments and collected the samples. YX and DH acquired, analyzed, and interpreted the data. JL and YC participated in data collection and analysis. All authors read and approved the final manuscript.

## Conflict of Interest

The authors declare that the research was conducted in the absence of any commercial or financial relationships that could be construed as a potential conflict of interest.
