# Supplementary material for: Pilose Antler Peptide-3.2KD Ameliorates Adriamycin-Induced Myocardial Injury Through TGF-β/SMAD Signaling Pathway
Source: Front Cardiovasc Med. 2021 May 28;8:659643. doi: 10.3389/fcvm.2021.659643 (PMC8194399; doi:10.3389/fcvm.2021.659643)
Supplement: Supplementary file 1 [file Table_1.DOC]

| **Table 1. Pathological score standard** | | | |
| --- | --- | --- | --- |
| Pathological area  ratio | Inflammation | Myocardial Fibrosis | Diffuse edema myocardial cells |
| None | 0 | 0 | 0 |
| <25% | 1 | 1 | 1 |
| 26–50% | 2 | 2 | 2 |
| 51–75% | 3 | 3 | 3 |
| >76% | 4 | 4 | 4 |
