# Supplementary material for: Pilose Antler Peptide-3.2KD Ameliorates Adriamycin-Induced Myocardial Injury Through TGF-β/SMAD Signaling Pathway
Source: Front Cardiovasc Med. 2021 May 28;8:659643. doi: 10.3389/fcvm.2021.659643 (PMC8194399; doi:10.3389/fcvm.2021.659643)
Supplement: Supplementary file 2 [file Data_Sheet_1.docx]

Fig1S

Molecular weight (MW) of PAP were measured using western Blotting. The results demonstrate that MW of PAP are 3.2KD and 10KD separately. In the present study, PAP with 3.2 KD was used for ADR-induced myocardial injury.


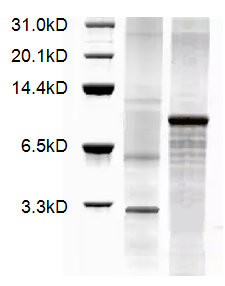


**10KD**

**3.2KD**

**3.3KD**

**6.5KD**

**14.4KD**

**20.1KD**

**31KD**
